# Strategies and Tools for Supporting the Appropriateness of Drug Use in Older People

**DOI:** 10.3390/ph15080977

**Published:** 2022-08-08

**Authors:** Carlotta Lunghi, Caterina Trevisan, Michele Fusaroli, Valentina Giunchi, Emanuel Raschi, Elisa Sangiorgi, Marco Domenicali, Stefano Volpato, Fabrizio De Ponti, Elisabetta Poluzzi

**Affiliations:** 1 Department of Medical and Surgical Sciences, University of Bologna, 40126 Bologna, Italy; 2 Centre of Studies and Research on Older Adults, University of Bologna, 40126 Bologna, Italy; 3 Department of Health Sciences, Université du Québec à Rimouski, Lévis, QC G5L 3A1, Canada; 4 Department of Medical Sciences, University of Ferrara, 44121 Ferrara, Italy; 5 Pharmacy Service, Local Health Authority of Ferrara, 44121 Ferrara, Italy

**Keywords:** older adults, appropriateness, medication adherence, digital health, adverse drug reactions, polypharmacy

## Abstract

Through this structured review of the published literature, we aimed to provide an up-to-date description of strategies (human-related) and tools (mainly from the digital field) facilitating the appropriateness of drug use in older adults. The evidence of each strategy and tool’s effectiveness and sustainability largely derives from local and heterogeneous experiences, with contrasting results. As a general framework, three main steps should be considered in implementing measures to improve appropriateness: prescription, acceptance by the patient, and continuous monitoring of adherence and risk-benefit profile. Each step needs efforts from specific actors (physicians, patients, caregivers, healthcare professionals) and dedicated supporting tools. Moreover, how to support the appropriateness also strictly depends on the particular setting of care (hospital, ambulatory or primary care, nursing home, long-term care) and available economic resources. Therefore, it is urgent assigning to each approach proposed in the literature the following characteristics: level of effectiveness, strength of evidence, setting of implementation, needed resources, and issues for its sustainability.

## 1. Introduction

With the ageing of the population, an increasing proportion of individuals are affected by more than one chronic condition, namely multimorbidity [1]. The treatment of different comorbidities often leads to the use of several medications. Thus, it is not unusual that an increasing proportion of older individuals is exposed to multiple medications, known as polypharmacy. Despite the lack of consensus on polypharmacy definition, researchers more often use this term to indicate the use of five (or ten) medications [2]. A global prevalence of polypharmacy of 32.1% was estimated in Europe when a definition of 5 or more medications was used [3]. Nevertheless, the prevalence of polypharmacy varies not only according to its definition or the type of assessment used but also the country, the setting and patients’ age group. Current self-reported prevalence rates in older adults aged 70 years and above from seven European centres ranged from 16.4% (in Geneva) to 60.8% (in Coimbra) [4]. Another study reported prevalence estimates from 26.3% to 39.9%, depending on the country [3]. Different estimates are also reported for Italy: 49% of Italian patients older than 65 received polypharmacy (at least 5 concomitant medications) and 11.3% excessive polypharmacy (at least 10 medications) [5], with a higher prevalence in Southern Italy [6,7].

The aging population and the availability of new medications for chronic conditions can explain the rise in polypharmacy in many developed countries. On the other hand, it is not negligible the impact of the pharmaceutical industry and pharmaceutical sales representatives on the prescribing patterns of physicians. A recent systematic review found that the interaction with the pharmaceutical industry (through its sales representatives) is likely to affect physicians’ prescribing behaviours and contribute to the irrational prescribing of different medications [8]. In this context, the concept of pharmaceuticalization has been introduced to emphasize the importance of pharmaceuticals and the pharmaceutical industry in modern life [9]. Pharmaceuticalization can also explain the rising pharmaceutical choices of purchasing and using medications bypassing physicians, through over-the-counter drugs, herbal medicines, supplements or even internet-purchased medications without prescriptions (i.e., opioids or drugs for erectile dysfunction) [10]. Medication-related problems have been exacerbated by the COVID-19 pandemic. Indeed, during the pandemic, physicians and other healthcare professionals were in short supply, and medication reviews and other “non-essential” services were delayed or suspended to reduce the spread of the disease [11,12]. The pandemic has also increased the use and misuse of some medications, such as antidepressants, benzodiazepines, or antipsychotics [13], and increased self-medication behaviours [14].

Despite the variability of polypharmacy estimates and the reasons underlying its rising, it is consistently reported that polypharmacy is associated with increased risk for drug-drug or drug-disease interactions, adverse effects, potentially inappropriate medications (PIMs), geriatric syndrome, falls, and mortality [15,16]. There is generally little guidance in treating multimorbidity in older adults. In Italy, Onder et al., have recently developed specific guidelines for managing individuals exposed to multimorbidity and polypharmacy [17]. These guidelines underline the importance of an individualized and multidisciplinary approach and identifying individuals at higher risk for adverse outcomes of polypharmacy, despite there being no evidence that the number of medications (polypharmacy), rather than inappropriate polypharmacy, is directly responsible for these adverse outcomes. Adverse drug reactions (ADR) are very frequent in geriatric patients: a meta-analysis estimates that ADRs are responsible for 8.7% (95% CI = 7.6–9.8%) of hospital admissions [18]. Non-steroidal anti-inflammatory drugs (NSAIDs) were among the most common classes related to hospital admissions, which ranged from 2.5% to 33.3% in the studies [19]. Other medications implicated in ADRs included beta-blockers (1.8–66.7%), antibiotics (1.1–22.2%), oral anticoagulants (3.3 to 55.6%), digoxin (1.6–18.8%), angiotensin-converting enzyme inhibitors (5.5–23.4%), oral antidiabetics (4.5–22.2%), and opioids (1.5–18.8%). Risk factors for ADR-related hospitalizations included the number of medications (in all the studies analyzing this variable), the number of comorbidities, female sex, age, and inappropriate medications. Therefore, reducing the number of prescriptions in older adults might improve health and reduce hospitalization and mortality [20,21]. In this context, interventions, strategies, and tools to minimize the iatrogenic risks for multimorbid older patients by reducing the number of drugs they take are strongly recommended.

In this paper, we aimed to give an up-to-date description of the strategies and tools supporting the appropriateness of drug use in older adults.

## 2. Diagnosis and Medical Prescription in Older Adults

The care pathways of older patients may substantially differ from those of their younger counterparts with the same disease, especially considering treatment options and choices. This is because the focus of geriatric medicine does not lie on the disease but on the whole individual. The main goal is not just the treatment of a pathologic condition but the maintenance as much as possible of self-sufficiency, social participation, and quality of life. Therefore, as recommended by the Italian guidelines for managing people with multimorbidity and polypharmacy, physicians should consider these patients’ health trajectories, needs, and preferences and set realistic therapeutic targets [17,22]. After diagnosis, the medical prescription process is primarily driven by the need to avoid the disease’s clinical manifestations and complications, including its interactions with coexistent conditions and pharmacologic therapies. This means that physicians should adjust the clinical recommendations reported in the guidelines for single diseases to each patient’s characteristics. A further crucial point concerns the need for a frequent re-evaluation of the ongoing treatment appropriateness. Indeed, since older individuals frequently have unstable health trajectories, single therapies’ risk/benefit ratios may vary with the changes in clinical conditions. Close follow-up assessments may also allow physicians to question the current patients’ needs and treatment goals and consider the introduction, maintenance, or discontinuation of different treatments [17]. Hospitalization is a delicate moment in the management of drug therapy in older adults. Mucalo et al., describe that nearly one-third of patients have a potentially inappropriate prescription at discharge [23]. In geriatric units, performing a medication review may reduce the number of potentially inappropriate prescriptions (PIPs) and the risk of iatrogenic events [24]. The experience of a German university hospital showed that the number of PIP observed six months after discharge was significantly reduced in patients with at least one in-hospital therapeutic reconciliation. Nevertheless, no difference was found between reconciliation during hospitalization or at discharge [25].

## 3. Medication Adherence

Once the most appropriate therapeutic approach for the patient is defined, physicians should dedicate adequate efforts and time to inform and share the care plan with the patient, their caregivers (e.g., family members or non-healthcare professionals taking care of an older individual who is sick or not able to take care of themself), and other healthcare professionals who play a role in their care process. Effective physician-patient interaction is a cornerstone to increasing the patient’s comprehension of medical recommendations [26] and facilitating the acceptance of the prescribed therapies. In this regard, extensive literature has evidenced that deep communication between physician and patient on diagnosis and prescriptions with shared decision-making improves adherence to the medical recommendations and short- and medium-term clinical outcomes [27,28,29]. Previous reports found that around half of the patients discharged from the emergency department would not be able to understand written medical recommendations completely [30,31,32,33]. In addition to patients’ and caregivers’ awareness of the need, role and possible adverse effects of the prescribed recommendations, acceptance may be influenced by other aspects after initiation of the treatment. Among these are the drugs’ beneficial effects on disease control and quality of life, the tolerability of the prescribed therapy [34], and the ease of administration in terms of drug formulations and dosage forms [35].

When dealing with older patients, maintaining a high level of adherence to the medical recommendations is still a challenge, especially among those coping with multimorbidity. In this population, previous reports estimated that the prevalence of medication adherence is only around 50% [36]. A crucial moment in the patient’s care pathway is represented by the transition between secondary and primary care. Indeed, after hospital discharge, patients and caregivers may experience difficulties following new or modified medical recommendations. In a study comparing treatments prescribed at hospital discharge and those actually taken at home after 48 h in a sample of individuals aged 70 years or older, researchers found discordances in 56% [37].

A crucial enabling factor of medication adherence is interpersonal trust between physician and patient, which is a vital aspect of the patient-physician relationship, particularly for older patients [38,39]. According to Thom et al., low trust in physicians is associated with poorer adherence to medical recommendations, lower satisfaction with care, and diminished symptoms’ improvement [40]. Moreover, trusting their physician leads patients to disclose their health-related behaviours, even those they believe are shameful [38]. Qualitative data suggest that patients’ trust in general practitioners is crucial to establishing positive beliefs and becoming willing to deprescribe medications after a medication review [41]. Trust in physicians and the pharmaceutical industry seems to have been worsened by the recent COVID-19 pandemic because of the rapid growth in contradictory information on the internet, social media and traditional media [42].

Poor medication adherence is a prevalent problem in older age because geriatric patients have a complex set of risk factors, including the presence of multiple chronic diseases that co-exist with cognitive and functional deficits. It is essential to make older patients or their caregivers able to report possible use of over-the-counter medications, adverse events, and difficulties in following prescription recommendations. Identifying people with potential risk factors for non-adherence would be a key step for prescribers and healthcare providers in order to focus efforts on supporting adherence to medications. For instance, previous reports have observed that the use of over-the-counter medications was influenced by sociodemographic factors (e.g., educational and socioeconomic levels), individual aspects (e.g., health literacy, disease experience), and policies in the local healthcare system [43].

The consequences of non-adherence to medical recommendations can occur in the short- and longer-term. Indeed, poor medication adherence has been associated with scarcer disease control, higher hospitalization needs, lower quality of life and shorter survival [44,45,46,47]. In light of the relevance and impact of this factor, several intervention studies have evaluated the best strategies to improve medication adherence in adult and older patients in different settings of care. These concerned educational, pharmacist-led, nurse-led, or reminder/simplification approaches [25,48,49] (see below). However, an integrated multidisciplinary approach with these strategies combined may provide the best solution to promote adherence to medical recommendations in older patients and to positively influence clinically relevant outcomes.

## 4. Strategies Supporting the Appropriateness of Medication Use

### 4.1. Prescriber’S Tools

#### 4.1.1. Lists and Indexes

An appropriate prescription refers to the proper medication treatment for the patient’s needs at the correct dose and the required duration. Many well-validated tools for evaluating the appropriateness of medications for older adults exist. A recent systematic review identified all the published tools to guide clinicians in optimizing drug treatment in older adults [50]. The most known are those based on lists of medications that should not be used (or that should be initiated) in older individuals, such as the Beers [51] or the Screening Tool to Alert to Right Treatment (START)/Screening Tool of Older Persons’ Prescriptions (STOPP) criteria [52]. These widely used criteria are based on expert consensus processes (i.e., Delphi) and are revised periodically based on new evidence. Many other consensus-based lists of medications have been developed. The majority are based on consensus-based lists of medications to be avoided in older adults (and, sometimes, necessary drugs) [53,54,55,56,57,58]. The medication appropriateness index (MAI) [59] is based on a list of structured questions (i.e., on the presence of an approved and/or evidence-based indication, an effective dosage, and the lack of duplication), without addressing specific drugs. It is often used during the medication review process. The Fit fOR The Aged (FORTA) list [60] has classified all the medications used to treat chronic diseases in older adults into four classes according to the evidence on the efficacy and safety, and the appropriateness for the age group.

Although many of these lists repose on similar evidence to build classes of medications to avoid in older adults, differences exist, and the prevalence of PIMs may vary widely depending on the tool used. A recent study comparing the European Union Eu(7)-PIM list and Beers and STOPP criteria showed poor concordance among these tools in identifying inpatients exposed to PIMs [61]. Moreover, the applicability in different settings and countries of these tools has been studied only for a few tools, such as Beers and START/STOPP criteria. Many other country-specific criteria have been proposed in recent years to improve applicability to the specific healthcare system, especially because of the absence of specific medications in the country-specific market. Examples in Europe are the REview of potentially inappropriate MEDIcation pr[e]scribing in Seniors (REMEDI[e]S) in France [62], the PRISCUS list in Germany [54], and the NORGEP-NH criteria in Norway [63]. The Eu(7)-PIM list has been developed through a consensus of experts from 7 different European countries (Denmark, Estonia, Finland, France, Netherlands, Spain, and Sweden) with the aim of clinical applicability in Europe. Nevertheless, the applicability of these criteria to all European countries is still limited, especially for the Eastern and Central European Countries [64]. It is, therefore, necessary to define all the better strategies to improve the appropriateness of drug use in older adults according to the specific country but also to the specific care setting. Differences from a regulatory, legal and cultural point of view have to be acknowledged to implement the use of these tools in routine clinical practice.

#### 4.1.2. Medication Review in Team

**Figure 1:**
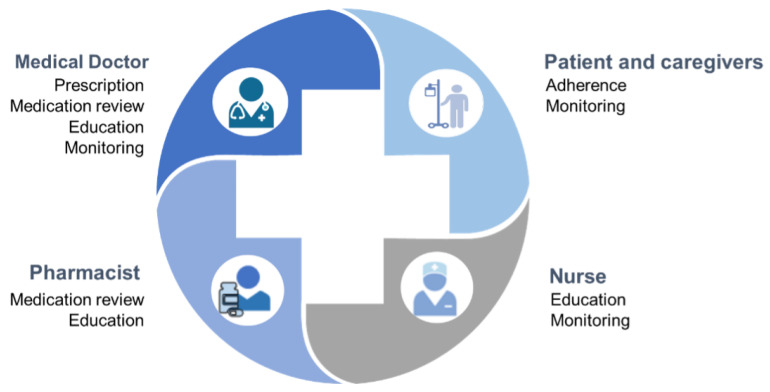
The shared effort toward appropriateness of drug use.

Although physicians have the main role and responsibility in prescribing medicines, optimization of drug use in older adults needs support from other healthcare professionals, especially for chronic therapies. The medication review process can be split into different steps, and trained nurses and clinical pharmacists may be active in some of these, with well-defined roles (Figure 1). At the discharge from the hospital, as well as after second or primary care access, when patients have to be aware of why, when, how much and how long to use prescribed medicines, both can support physicians in verifying patient and caregiver awareness and therefore in promoting compliance. Again, both professionals can be enrolled in monitoring adherence and some endpoints of the risk-benefit profile during the therapy, even without the direct involvement of the physician, if appropriate local services are arranged.

Nevertheless, a crucial point is interprofessional collaboration. General practitioners (GPs) represent most older individuals’ principal contact with healthcare professionals, as they regularly monitor symptoms and oversee refilling prescriptions. However, specialists manage patients with chronic diseases and are often in charge of adding or stopping medicines for these conditions without consulting GPs. Many experiences of pharmacist-led service have been described in the literature, and its optimization represents a current challenge. Successful pharmacist interventions are regular consultation (for instance, at the time of prescription refill) for detecting possible drug-drug interactions and adverse drug reactions, strengthening education and the importance of adherence, as well as supporting the use of apps of reminders and, when feasible, providing the patient with personalized pillboxes (see below). Focusing on specific cohorts of patients (e.g., with diabetes or oncological diagnoses) seems to increase the impact on outcomes, especially for process endpoints, as the number of concomitant medications and adherence [65]. Nurse-led initiatives have been especially focused on specific cohorts of patients, for which specialized healthcare professionals are needed: medication self-management training programs for chronic psychiatric treatment and patient-navigator service for oral oncological therapies are currently promising topics [66,67]. The collaboration between GPs, specialists, pharmacists, and nurses enables the effective implementation of medication review in clinical practice. Nonetheless, the opportunity to work as a team for different healthcare professionals involved in the medication review process necessitates adjusting the current clinical practice for shared decision-making. Moreover, the economic sustainability and impact on clinical outcomes of each strategy are not yet strongly demonstrated [68].

#### 4.1.3. Electronic Tools Supporting Appropriate Prescription

Current prescribing practice is frequently supported by electronic tools, which allow doctors to simultaneously include each prescription into the patient’s electronic health record and provide them with their receipt. This habit urges specific computerized prescription support systems to help medication review and therapeutic choice, especially for patients with comorbidity and polypharmacy. These systems belong to the larger area of digital health interventions (DHIs), which are technologies facilitating the accomplishment of the health needs of individuals and populations, and include e-Health (e.g., informative websites, educational videogames, telehealth webinars) [69,70], and m-Health (e.g., mobile microsensors, apps to study voice markers) [71].

The potential role of DHIs is broad and not yet fully explored. Many online resources are available for physicians, from authoritative websites, such as deprescribing.org, which provide recommendations, videos and list useful apps for specific therapeutic areas and users, to software with the relevant app for computer or smartphone. They may be used for single cases during the visit or integrated with the electronic chart databases and used to automatically receive a warning on potential inappropriate prescriptions or to process all single patient prescription lists periodically. Some examples of these specific websites are medstopper.com, drugs.com [folder: interaction checker], and intercheckweb.marionegri.it (in Italian). As for integrating DHIs in the electronic chart, a typical example is represented by platforms that document and track patients’ therapy and clinical conditions (e-medication history). Some of them put a red flag close to potential interactions and remember to prescribe the investigations for early detection of adverse reactions over the follow-up (e.g., lipidaemia in antipsychotics).

#### 4.1.4. Web Resources on Adverse Reaction Prevention

Concerning side effects of medicines, digital tool development is strongly focused on their prevention and early detection. Drug-induced Torsades de Pointes (TdP) and Drug-Induced Liver Injury (DILI) are among the most frequent causes of drug attrition during drug development and drug withdrawal in the post-marketing setting [72,73]. These adverse drug reactions share several similarities; though erroneously considered idiosyncratic, they actually occur in a dose-dependent manner in subjects with several host- and patient-related risk factors [74,75]. For instance, atrial fibrillation and previous myocardial infarction, which are highly prevalent in older adults, represent typical risk-factor for developing TdP in case of multiple drug treatments with antiarrhythmics, antipsychotics and some specific antimicrobials. Dedicated websites (www.crediblemeds.org, accessed on 27 July 2022; for TdP) and bookshelves (https://www.ncbi.nlm.nih.gov/books/NBK547852/ accessed on 27 July 2022; for DILI) have been implemented to support researchers and prescribers in therapy optimization, namely risk assessment in the individual patient [76].

Notwithstanding these efforts, our understanding, prediction and prevention in clinical practice are still unsatisfactory, especially for DILI, where the mechanistic basis and the *primum movens* are still uncertain. In this scenario, the question arises as to whether digital tools can actually support appropriateness, especially in older adults, or, conversely, are disregarded by clinicians due to alert fatigue.

With regard to TdP, cardio-oncology is an emerging rapidly-evolving area where a proactive medication review should be targeted as a preventive strategy to counteract the so-called reduced repolarization reserve caused by multiple drugs possibly interacting through pharmacokinetic and pharmacodynamic mechanisms [77]. A recent systematic review analyzed the use of risk assessment tools (RATs), including risk scores, computerized physician order entry systems and clinical decision support systems, as a strategy to identify patients at risk of TdP for repetitive or continuous electrocardiogram monitoring, discontinuation of pro-arrhythmic drugs, or serum electrolyte concentration monitoring [78]. The various RATs have peculiarities, including the heterogeneous setting of use and validation (e.g., intensive care units, hospital wards with different specialties). They are still suboptimal in terms of predictive performance, thus making combined use of RATs a candidate approach to reduce unnecessary alerts. Future studies are warranted to verify the potential adaptation in the outpatient setting and assess the actual impact on these DHIs, especially on hard endpoints such as hospitalization.

Regarding DILI, there are no recognized predictive DHIs. The opportunity for a medication review and stringent monitoring of transaminases remains pivotal strategies to reduce the burden of (inappropriate) co-medications and perform a timely diagnosis on a case-by-case basis. In this context, considering DILI diagnosis requires careful exclusion of alternative (non-pharmacological) causes, Hayashi et al., recently updated, simplified and computerized the Roussel Uclaf Causality Assessment Method (RUCAM), a current standard diagnostic algorithm, and developed an electronic evidence-based version, called RECAM, which is a promising user-friendly practice-changing tool also for clinicians without consolidated experience in DILI diagnosis [79]. Although further validation and refinement of criteria are needed, RECAM is an additional step in the era of digital medicine. It can also be implemented by adding pharmacokinetic substantiation to support the underlying pharmacological basis.

### 4.2. Patient’s Tools

#### 4.2.1. Digital Tools for the Patient

Medicine digitalization also represents an opportunity for the patients. Some DHIs may primarily target the patients or their caregivers, indeed. For example, they may remind the patient that a pill should be taken at a specific time or make more accessible information included in the package insert or the electronic healthcare record. Many new mobile applications focus on monitoring body parameters using microsensors (e.g., physical activity, blood pressure, vocal markers). They may facilitate communications between patients and physicians, for example, reporting suspect adverse reactions, adherence information, and vital signs parameters directly to the electronic healthcare record.

Nonetheless, the heterogeneity and diversity of the available DHIs make their choice difficult for unsupervised patients. In 2021, an extended search of the Apple and Google Play Stores for apps conceived to increase medication adherence found more than 2000 heterogeneous, mostly uncertified, mobile applications [80]. To drive the systematization of digital health, the WHO implemented the classification of digital health interventions (DHIs, version 1.0), distinguishing between different users and functions. However, this classification was targeted at app developers and not the patients, so the difficulties met when choosing a DHI remain.

A second problem concerns the accessibility to older adults: most the DHIs, especially those not specifically designed for older adults, are poorly accessible to them [81]. But digital interventions specifically thought for primary prevention in older adults have been developed, including tools to gather health data for goal planning, video consults and online webinars [70].

In the attempt to drive the development of more accessible and effective interventions, Matthew-Maich et al., performed a scoping review to collect lessons specific for designing, implementing, and evaluating mHealth support for older adults at home and their caregivers [82]. Currently, many DHIs are characterized by low scientific quality and patient appreciation, and Backes et al., concluded that none of the more than 2000 apps investigated should be recommended by health providers [80]. Following accruing lessons –focusing on motivation (goal-setting and rewards), remote help, support by other patients, feedback by healthcare providers, and accessibility (native language and printable material) [70], will plausibly result in higher adherence. In particular, when addressing older adults population, it is of the upmost importance to account for digital inequalities related to sociodemographic gaps, in informatics skills and resources, together with cognitive decline and visual impairment [83].

Another promising option concerns the possibility of developing apps that allow information sharing among different stakeholders, providing role-specific interfaces. The same app, for example, may automatically remind the patients to take their drug, alert the caregiver in case of omission, and show the adherence interpolated with biomarker data in the electronic healthcare record for the physician (e.g., showing the relationship of the blood pressure of the patients and their adherence to antihypertensive drugs). Further, future apps may be personalized based on the patient’s health conditions and the setting. For example, for non-compliant patients, it may be advisable to document to the caregiver the drug assumption by recording it on a video.

Finally, it is easier to develop effective interventions if specific populations are targeted, for example, patients with frailty (e.g., cognitive impairment, disability, chronic conditions) [71].

#### 4.2.2. Dose-Dispensing Tools

As mentioned above, poor medication adherence is a common issue for older individuals. Even when the patient has accepted their treatment and the communication between healthcare professionals and the patient is good (see below), unintentional non-adherence may still occur. It occurs indeed when the patients forget to take their medications or they do not well understand the provider’s indications [84]. Sometimes, especially in older adults, unintentional non-adherence occurs because of physical, mental, or psychological barriers leading to the inability to manage their treatment [85]. A peculiar problem is the complexity of the therapeutic regimen, a common issue in patients with multimorbidity and polypharmacy.

Dose-dispensing services are especially useful for older patients experiencing unintentional non-adherence [86]. The purpose of dosing aids is thus to assist patients in taking their medications and improve their adherence to medication [87]. These dose-dispensing tools (also known as dosettes or pillboxes) are storage devices for oral medications that also serve as a medication aide-mémoire to remind patients to take their medications at the right time [88,89]. The simplest ones have seven compartments for each day of the week, but they come in different sizes and shapes with subcompartments for different times of the day [87]. They can be filled by physicians, pharmacists, nurses or even by patients themselves or their caregivers [90]. In addition to making medication self-administration easier, these tools can improve unintentional adherence caused by forgetfulness and confusion [91,92,93]. These services are commonly implemented in hospitals and community pharmacies in many countries In an effort to better support patients, families, and caregivers, with new technologies, new dose-dispensing tools (smart pill dispensers) have been developed to help manage complex pharmacotherapies, such as pillboxes with visual and sound alarms that will alert the user to take the medication at the time it must be taken, or other sending email or notifications if a dose is skipped or taken at the wrong time [94]. Nevertheless, despite new technologies and tools being implemented in recent years, all dose-dispensing services should be subject to a medication review at the beginning and repeated regularly. Moreover, patient communication and coaching should accompany these tools or services with close cooperation among all actors involved in patient care (physicians, nurses, pharmacists, and caregivers) [95]. Table 1 summarizes the main tools available to support the appropriateness of drug utilization in older individuals.

## 5. Communication between Physician and Patient

As anticipated above, communicating with the patient is the first step to ensuring high adherence to medical recommendations. The term communication comes from Latin, and its original meaning is “to share”. In the physician-patient interaction, there is a bidirectional sharing, not only of medical information from the physician to the patient but also of doubts and experiences from the patient to the physician. However, not always adequate attention is paid to this issue in daily clinical practice.

In an interesting study evaluating the interface between physicians and patients, only around 20% of patients had the opportunity to fully explain their concerns. In comparison, in almost 70% of cases, physicians prematurely interrupted the open statement of the patient to direct specific questions [96].

In line with this result, other primary or secondary care studies found that physicians tend to interrupt patients after a median time ranging from 11 to 23 s [97,98]. Conversely, giving them the possibility to fully express their concerns without interruptions would have only taken up to two minutes and given physicians most of the needed information [97]. Although physicians may be reluctant to ask open-ended questions due to limited time to dedicate to the visits, leaving patients free to express their concerns and open statements seems to be the most appropriate strategy and may limit the loss of useful information to drive physicians’ diagnosis and treatment choices. Adding leaflets and online educational programs can further improve patient awareness and empowerment.

As far as physician-related factors influencing communication with the patient are concerned, some sociodemographic characteristics have shown to play substantial roles. For instance, in primary and secondary care, female physicians tended to spend approximately two minutes more than men in medical visits and establish a more patient-centered communication [99], with a higher emotional involvement [100]. Ethnicity may be an additional factor influencing some communication aspects. A previous work conducted in the United States found that patients undergoing a medical visit with a physician of the same ethnicity were more satisfied and perceived higher physician participation than those undergoing an ethnicity-discordant visit [101]. The length of work experience might also interfere with the ability to spend enough time with the patient. In a study focused on the communication of bad news to older patients, in fact, physicians with a longer work experience appeared to be more likely to dedicate an adequate amount of time to talk with the patient than those with fewer working years [100].

Considering patient-related factors influencing communication, poor health literacy could affect the comprehension of the medical recommendations. This aspect is particularly important for older people affected by multiple chronic conditions, who often take several drugs. In a study evaluating medication errors reported by patients, almost 80% of the involved patients reported at least one mistake, and in most cases, errors were due to difficulties in identifying the medication or understanding medical instructions. The presence of multiple chronic diseases, multidrug regimens, and changes in medical prescriptions emerged as factors associated with the probability of reporting medication errors [102]. In addition, older patients frequently suffer from conditions that can impair their ability to understand and correctly follow medical recommendations, such as vision or hearing impairments, cognitive deficits, or mobility restrictions [103]. The physician should recognize these problems and adapt the communication style to overcome these possible obstacles to a correct understanding of medical recommendations.

Identifying and acting on the factors that may affect the communication with the patient is of great relevance in consideration of the impact of such aspects on several health-related outcomes. First of all, enhancing communication between patients and healthcare professionals is the key to ensuring that patient preferences are taken into consideration, thereby patient adherence and their experience are improved [104]. This means exposing the patient to a lower risk of having new hospitalizations for unbalances of chronic diseases, poor quality of life and mortality, and reducing the costs for the healthcare systems [44,46,47,105]. A crucial moment to ensure good physician-patient communication is the transition between different care settings, such as hospital discharge. In this context, discrepancies between the usual and new prescribed therapies, in the absence of adequate communication and explanation of the medical recommendations, could predispose to medication errors and poor adherence. Although trials on the effectiveness of educational/behavioural and reminder/simplification interventions have given promising results, further investigations should be devoted to undercover the patients’ point of view of communication on medical recommendations, in specific care settings or during transitions between them [106].

## 6. Conclusions

Physicians, especially geriatricians, strongly agree on the importance of periodic medication reviews and their advantage for the patient in terms of adherence as well as overall health outcomes. It’s also established that other healthcare professionals, namely pharmacists and nurses, should be included in the medication review process, maintaining their roles and supporting both patients and prescribers. Digital health interventions represent useful solutions to help professionals identify inappropriate prescriptions and maintain patient adherence to prescribed therapies. The efficacy of every single strategy is far from being well demonstrated, especially in terms of main clinical outcomes and health and economic sustainability. However, the need for integrated strategies is largely shared among physicians, patients and policymakers.

Identifying the most appropriate approach requires defining the specific setting of care (hospital, ambulatory or primary care) and if a particular cohort of patients should be prioritized.

As a general framework, three main steps should be considered in implementing measures to improve appropriateness: prescription, acceptance by the patient, and continuous monitoring of adherence and the risk-benefit profile. Each step needs efforts from specific actors (doctors, patients, caregivers, healthcare personnel) and dedicated supporting tools. Moreover, how to support the appropriateness also strictly depends on the particular setting of care (hospital, ambulatory or primary care) and available economic resources. Therefore, it is urgent assigning to each approach proposed in the literature the following characteristics: level of effectiveness, the strength of evidence, setting of implementation, needed resources, and issues for its sustainability.

## Figures and Tables

**Table 1:** Main tools supporting the appropriate drug use.

Tools	Description	Examples
**Prescriber’s tools**		
**Lists and indexes**	Lists of medications to be or not to be used based on efficacy, safety, and appropriateness	Beers criteria; START/STOPP * criteria; FORTA * list; REMEDI[e]S *; PRISCUS list; NORGEP-NH criteria; Eu(7)-PIM list
	General appropriateness indexes	MAI *
**Electronic tools**	DHIs * providing recommendations, videos, and apps for specific therapeutic areas	www.deprescribing.org, accessed on 27 July 2022
	Websites and bookshelves supporting patients and prescribers in therapy optimization	www.medstopper.com, accessed on 27 July 2022; www.drugs.com, accessed on 27 July 2022 [folder: interaction checker]; www.intercheckweb.marionegri.it, accessed on 27 July 2022
**Web resources on adverse drug reactions**	Websites and bookshelves supporting patients and prescribers in therapy optimization	www.crediblemeds.org, accessed on 27 July 2022; www.ncbi.nlm.nih.gov/books/NBK547852/, accessed on 27 July 2022
	RATs * and diagnostic algorithms to identify patients at risk for adverse reactions	Risk scores; computerized physician order entry systems; clinical decision support systems; RECAM *
**Patient’s tools**		
**Digital tools for the patients**	Mobile applications facilitating communications between patients and physicians	Apps reporting suspect adverse reactions, adherence information, and vital signs parameters directly to the electronic healthcare record
	DHIs * helping patients and caregivers adhering to treatment	Apps reminding the patient that a pill should be taken at a specific time; apps making more accessible information included in the package insert
	DHIs * for information sharing among different stakeholders	Apps that remind the patient to take pills, alert the caregiver in case of omission, and show the adherence interpolated with biomarker data in the electronic healthcare record for the physician
**Dose-dispensing tools**	Dose-dispensing services for patients experiencing unintentional non-adherence	Pillboxes with seven compartments for each day of the week; pillboxes with visual and sound alarms

* DHIs: digital health interventions; FORTA: Fit fOR The Aged; MAI: Medication appropriateness index; RATs: risk assessment tools; RECAM: Revised Electronic Causality Assessment Method; REMEDI[e]S: REview of potentially inappropriate MEDIcation pr[e]scribing in Seniors; START/STOPP: Screening Tool to Alert to Right Treatment/Screening Tool of Older Persons’ Prescriptions.

## Data Availability

Not applicable.
